# Comparison of gene set scoring methods for reproducible evaluation of tuberculosis gene signatures

**DOI:** 10.1186/s12879-024-09457-z

**Published:** 2024-06-20

**Authors:** Xutao Wang, Arthur VanValkenberg, Aubrey R. Odom, Jerrold J. Ellner, Natasha S. Hochberg, Padmini Salgame, Prasad Patil, W. Evan Johnson

**Affiliations:** 1 Department of Biostatistics, Boston University, Boston, MA USA; 2 Division of Computational Biomedicine and Bioinformatics Program, Boston University, Boston, MA USA; 3 Division of Infectious Disease, Center for Data Science, Rutgers New Jersey Medical School, Newark, NJ USA; 4 Department of Medicine, Center for Emerging Pathogens, Rutgers New Jersey Medical School, Newark, NJ USA; 5 Boston Medical Center, Boston, MA USA; 6 Section of Infectious Diseases, Boston University School of Medicine, Boston, MA USA

**Keywords:** Gene scoring methods, Original model, Reproducibility

## Abstract

**Background:**

Blood-based transcriptional gene signatures for tuberculosis (TB) have been developed with potential use to diagnose disease. However, an unresolved issue is whether gene set enrichment analysis of the signature transcripts alone is sufficient for prediction and differentiation or whether it is necessary to use the original model created when the signature was derived. Intra-method comparison is complicated by the unavailability of original training data and missing details about the original trained model. To facilitate the utilization of these signatures in TB research, comparisons between gene set scoring methods cross-data validation of original model implementations are needed.

**Methods:**

We compared the performance of 19 TB gene signatures across 24 transcriptomic datasets using both rrebuilt original models and gene set scoring methods. Existing gene set scoring methods, including ssGSEA, GSVA, PLAGE, Singscore, and Zscore, were used as alternative approaches to obtain the profile scores. The area under the ROC curve (AUC) value was computed to measure performance. Correlation analysis and Wilcoxon paired tests were used to compare the performance of enrichment methods with the original models.

**Results:**

For many signatures, the predictions from gene set scoring methods were highly correlated and statistically equivalent to the results given by the original models. In some cases, PLAGE outperformed the original models when considering signatures’ weighted mean AUC values and the AUC results within individual studies.

**Conclusion:**

Gene set enrichment scoring of existing gene sets can distinguish patients with active TB disease from other clinical conditions with equivalent or improved accuracy compared to the original methods and models. These data justify using gene set scoring methods of published TB gene signatures for predicting TB risk and treatment outcomes, especially when original models are difficult to apply or implement.

**Supplementary Information:**

The online version contains supplementary material available at 10.1186/s12879-024-09457-z.

## Introduction

Tuberculosis (TB) is the leading infectious cause of death worldwide [1, 2]. Approximately 10 million people develop TB, and 1.4 million die from the disease [1]. Current microbiological diagnostic tests for TB disease include sputum acid-fast bacilli (AFB) smear microscopy, rapid molecular tests, and culture-based technologies [1]. With the advent and widespread availability of nucleic acid amplification tests (Xpert MTB/RIF), some cases of pulmonary TB can be diagnosed quickly and accurately. Paucibacillary TB diagnosis (smear-negative pulmonary, extrapulmonary, and pediatric TB) [3–6] and predicting treatment success/failure remain difficult challenges. Furthermore, there are gaps in identifying individuals with slow-growing quiescent or percolating disease, and utilizing based technologies would facilitate diagnosis for individuals unable to produce sputum (e.g., children). There is therefore an urgent need for additional technologies that ensure high-quality, timely, effective testing for people living with TB [1, 7].

Multiple blood-based biomarkers have been developed for TB over the past ten years. These signatures can distinguish active TB disease from latent TB infection (LTBI) [8, 9], distinguish TB from other diseases [10–12], predict progression from LTBI to active TB [13, 14]. They may meet target product profiles proposed by the World Health Organization for point-of-care testing [13–16]. However, more research must be done to establish the efficacy and reproducibility of using blood-based signatures in the field, as shown by the CORTIS trial, where the gene expression profile fails to predict downstream treatment/outcome of TB [17].

In the case of existing TB signatures, the *replicability* of these biomarkers is inadequate, meaning that many of the original publications did not give enough detail to replicate the published models. Some of these original models further lacked *reproducibility* of the accuracy of TB gene signatures—meaning that the signatures were *overfit* and thus experienced significant reductions in performance in later observations [10, 18, 19]. Several research teams have attempted to address these issues, either by rebuilding the original classification models [7, 18] or by using methods such as gene set enrichment analysis (GSEA) [20, 21].

Our team has recently released TBSignatureProfiler software, which provides a compilation of TB gene sets used from published biomarkers and provides methods [20] to evaluate the performance of these gene sets [22]. However, while alternative methods, such as gene set scoring, are simpler to use than the original models, these methods have not been established as reasonable approximations original model performance. To address the issues of reproducibility in reconstructing the discovery set, our study uniformly evaluated the performance of 19 TB gene signatures across 24 datasets using both original models and gene set scoring methods. We also curated the datasets used in this study and included the corresponding discovery model for each gene signature in the TBSignatureProfiler R package, enabling the reproducibility of all results.

## Methods

### TB gene signatures and gene set scoring methods

Nineteen existing TB gene signatures were selected for this study based on the results of Warsinske et al. to make a fair comparison of the performance of these signatures (Table 1) [10, 12–14, 23–32]. Additional details on the gene signatures and original diagnostic models used for comparison are provided in the online data supplement. Five gene set scoring methods, single sample GSEA (ssGSEA) [33], gene set variation analysis (GSVA) [22], pathway level analysis of gene expression (PLAGE) [34], Zscore [35], and Singscore (unidirectional and bidirectional versions) [36], were selected to evaluate the accuracy of TB gene signatures that distinguish active TB from other clinical conditions across 24 studies. Details on the datasets used for comparison can be found in the online data supplement. Moreover, the ‘biomarker splitting’ strategy for gene signatures was proposed to overcome the limitations of using methods including GSVA, ssGSEA, and Singscore, where the signatures were evaluated based on their upregulated and downregulated subsets (see the online data supplement for details on biomarker splitting strategy).

**Table 1:** Summary of TB gene signatures compared in the study (see supplementary materials for detailed dataset descriptions)

Signature Name	Gene Number	Comparison	Datasets	Original Model Description
Sweeney_OD_3	3	Active tuberculosis vs. (LTBI & HCs & OD)	GSE19491 & GSE42834 & GSE37250	Difference of geometric means between up and downregulated genes
Jacobsen_3	3	Active tuberculosis vs. LTBI	GSE19491	Linear Discriminant Analysis
LauxdaCosta_OD_3	3	Active tuberculosis vs. OD	GSE42834	Random Forest
Maertzdorf_4	4	Active tuberculosis vs. HCs	GSE74092	Random Forest
Sambarey_HIV_10	10	Active tuberculosis vs. OD	GSE37250	Linear Discriminant Analysis
Verhagen_10	10	Active tuberculosis vs. (LTBI & HCs)	GSE41055	Random Forest
Maertzdorf_15	15	Active tuberculosis vs. HCs	GSE74092	Random Forest
Leong_24	24	Active tuberculosis vs. LTBI	GSE10175	Ridge Logistic Regression
Kaforou_27	27	Active tuberculosis vs. OD	GSE19491	Difference of arithmetic means between up and downregulated genes
Anderson_42	42	Active tuberculosis vs. LTBI	GSE39940	Difference of sums between up and downregulated genes
Kaforou_OD_44	44	Active tuberculosis vs. OD	GSE19491	Difference of arithmetic means between up and downregulated genes
Anderson_OD_51	51	Active tuberculosis vs. OD	GSE39940	Difference of sums between up and downregulated genes
Kaforou_OD_53	53	Active tuberculosis vs. OD	GSE19491	Difference of arithmetic means between up and downregulated genes
Berry_OD_86	86	Active tuberculosis vs. OD	GSE19491	K-nearest neighbors algorithm
Bloom_OD_144	144	Active tuberculosis vs. (HCs & OD)	GSE42834	Support Vector Machines
Berry_393	393	Active tuberculosis vs. (LTBI & HCs)	GSE19491	K-nearest neighbors algorithm
Suliman_RISK_4	4	Incipient tuberculosis vs. HCs	GSE94438	Support Vector Machines (linear kernel, using paired ratio)
Zak_RISK_16	16	Incipient tuberculosis vs. HCs	GSE79362	Support Vector Machines (linear kernel)
Leong_RISK_29	29	Incipient tuberculosis vs. HCs	GSE79362	Lasso Logistic Regression

### Statistical analysis

The AUC value for each TB gene signature (sample scores against disease subtypes) was calculated for each dataset. The sample-size-weighted mean AUC (weighted AUCs) was used to assess the overall performance of each gene set across all studies while excluding the discovery dataset(s) used to train the corresponding signature [18].

Several metrics were used to compare the performance of gene signatures as assessed by different gene set scoring methods and their original models. For each TB gene signature, Spearman’s rank correlation ($\rho$) was computed to measure the strength of association of the prediction scores from a signature’s original model and the different gene set scoring methods. We then summarized the correlation results by computing the weighted Spearman’s rank correlation (${\rho }_{w}$), as outlined in Eq. 1, where ${n}_{i}$is the number of observations corresponding to study $i$.

1 $${\rho }_{w}=\frac{{\sum }_{i=1}^{k}{\rho }_{i}\text{*}{n}_{i}}{{\sum }_{i=1}^{k}{n}_{i}}$$

Moreover, we determined the absolute difference in AUC ($\left|\varDelta AUC\right|$) between the original model and various gene set scoring methods, as showed in Eq. 2.1 for each selected dataset. The weighted absolute AUC difference ${\left|\varDelta AUC\right|}_{w}$ was the calculated to represent the overall distribution pattern across all selected studies (Eq. 2.2). Additionally, the Szymkiewicz–Simpson coefficient [37], also known as the overlap coefficient ($oc$; Eq. 3) was applied to evaluate the similarity of studies based on the results given by each gene set scoring and original model for each biomarker.

2.1 $$\left|\varDelta AU{C}_{i}\right|= \left|AU{C}_{GSEA,i}-AU{C}_{original\; model,i}\right|$$

2.2 $${\left|\varDelta AUC\right|}_{w}=\frac{{\sum }_{\text{i}=1}^{k}\left|\varDelta AU{C}_{i}\right|*{n}_{i}}{{\sum }_{i=1}^{k}{n}_{i}}$$

3 $$oc=\frac{\# of \;common\; datasets}{min\left({n}_{GSEA},{n}_{original \;model}\right)}$$

Finally, code for the analyses from this paper can be found at: https://github.com/xutao-wang/Comparison-of-existing-tuberculosis-gene-signatures.

## Results

### Evaluation of gene signatures using discovery studies

The list of TB gene signatures and information on their training data are presented in Table 1 and online data supplement. The AUC values for our reconstructed models were nearly identical to the results of the original publications, suggesting that the training models were accurately reconstructed (Table S1). Several gene signatures including Sweeney_OD_3, Maertzdorf_15, Leong_24, Kaforou_27, Anderson_42, and Berry_393, when evaluated by ssGSEA, had estimated AUC values above 0.9 (Table S1). These results suggest that ssGSEA is a comparable signature profiling method, producing accurate results for some TB signatures in differentiating TB disease states.

### Performance of original models and gene set scoring methods

**Fig. 1:**
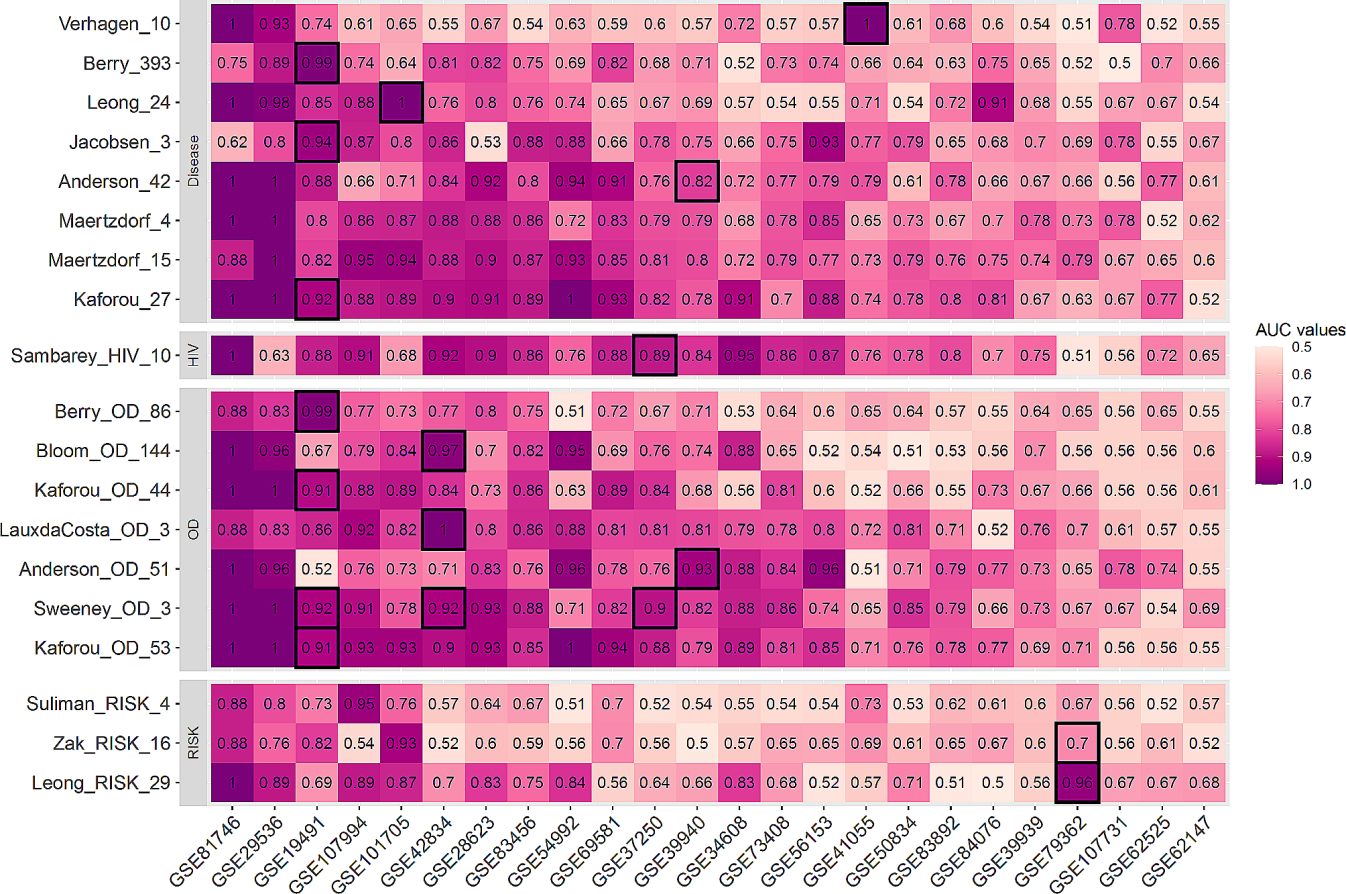
Heatmap of AUC distribution for each signature across 24 studies using the original model. Grids with black borders indicated the discovery sets for each TB gene signature. Each row represented one signature. Signatures were clustered into different categories according to the TB subtypes they identified. The column of the heatmap corresponded to the studies used in this paper. The datasets were rearranged in decreasing order based on their mean AUC values across all TB gene signatures

When TB gene signatures were evaluated by their original diagnostic model, thirteen of 16 signatures had AUCs greater than 0.9 from their discovery dataset(s) (Fig. 1). Notably, Kaforou_OD_53, Kaforou_27, Maertzdorf_15, and Sweeney_OD_3 had consistently high AUC values across different studies (> 0.8 weighted AUCs for all four gene sets; Table 2). In contrast, Verhagen_10 had a weighted AUCs of 0.61 (Table 2), performing well in some datasets (> 0.9 AUC in GSE81746, GSE41055, and GSE29536) but with poor performance in most of the remaining studies (< 0.65 AUC in 16 out of 24 studies; Fig. 1). Additionally, Zak_RISK_16, Suliman_RISK_4, and Leong_RISK_29 also performed poorly in these comparisons, but these are signatures of disease progression (Fig. 1).

**Table 2:** Weighted mean AUC and 95% CI for 19 gene signatures using the original model and gene set scoring methods (ssGSEA, GSVA, PLAGE, Zscore, and Singscore) across 24 studies

Signature	Warsinske et al.	TBSignatureProfiler (Original Model)	TBSignatureProfiler (ssGSEA)	TBSignatureProfiler (GSVA)	TBSignatureProfiler (PLAGE)	TBSignatureProfiler (Zscore)	TBSignatureProfiler (Singscore)
LauxdaCosta_OD_3	0.76 (0.45–1.00)	0.80 (0.76–0.83)	0.82 (0.76–0.86)	0.79 (0.74–0.83)	0.83^*^ (0.79–0.86)	0.78 (0.74–0.80)	0.82 (0.75–0.86)
Jacobsen_3	0.83 (0.69–0.98)	0.76† (0.71–0.80)	0.72 (0.68–0.76)	0.69 (0.64–0.73)	0.78 (0.75–0.81)	0.69 (0.65–0.73)	0.71 (0.67–0.75)
Sweeney_OD_3	0.85 (0.72–0.99)	0.81 (0.76–0.85)	0.82 (0.77–0.86)	0.75 (0.69–0.80)	0.82 (0.78–0.85)	0.77 (0.71–0.82)	0.82 (0.77–0.86)
Maertzdorf_4	0.79 (0.64–0.95)	0.80 (0.77–0.83)	0.70^*^ (0.65–0.75)	0.73^**^ (0.69–0.78)	0.81 (0.78–0.85)	0.72^**^ (0.68–0.76)	0.66^*^ (0.62–0.72)
Verhagen_10	0.54 (0.41–0.68)	0.61‡ (0.57–0.66)	0.57 (0.55–0.60)	0.59 (0.56–0.64)	0.65 (0.59–0.71)	0.62 (0.57–0.68)	0.58 (0.55–0.61)
Sambarey_HIV_10	0.82 (0.57–1.00)	0.83 (0.76–0.87)	0.80 (0.76–0.84)	0.80 (0.76–0.84)	0.76 (0.70–0.83)	0.75 (0.70–0.81)	0.79 (0.75–0.83)
Maertzdorf_15	0.79 (0.66–0.92)	0.82 (0.79–0.85)	0.83 (0.79–0.87)	0.82 (0.78–0.86)	0.83 (0.80–0.86)	0.79 (0.75–0.82)	0.79 (0.75–0.84)
Leong_24	0.75 (0.54–0.95)	0.72 (0.67–0.78)	0.73 (0.70–0.77)	0.61 (0.58–0.65)	0.73 (0.67–0.79)	0.61 (0.58–0.65)	0.73 (0.69–0.78)
Kaforou_27	0.83 (0.64–1.00)	0.81 (0.77–0.85)	0.82 (0.78–0.85)	0.79 (0.76–0.82)	0.83 (0.79–0.87)	0.79 (0.76–0.82)	0.78 (0.73–0.82)
Anderson_42	0.82 (0.66–0.97)	0.78 (0.73–0.83)	0.61^**^ (0.58–0.66)	0.60^**^ (0.58–0.64)	0.76 (0.70–0.82)	0.65^**^ (0.59–0.73)	0.57^**^ (0.53–0.61)
Kaforou_OD_44	0.78 (0.56–1.00)	0.76 (0.69–0.81)	0.67 (0.63–0.71)	0.72 (0.68–0.74)	0.80 (0.72–0.86)	0.70 (0.67–0.74)	0.70 (0.67–0.74)
Anderson_OD_51	0.58 (0.33–0.82)	0.71‡ (0.64–0.78)	0.75 (0.71–0.80)	0.81 (0.77–0.84)	0.79 (0.72–0.85)	0.75 (0.66–0.82)	0.77 (0.73–0.80)
Kaforou_OD_53	0.84 (0.70–0.99)	0.83 (0.78–0.87)	0.70^**^ (0.66–0.75)	0.77^**^ (0.74–0.80)	0.84 (0.80–0.87)	0.77^**^ (0.73–0.80)	0.77^**^ (0.73–0.81)
Berry_OD_86	0.69 (0.36–1.00)	0.69 (0.66–0.72)	0.71 (0.68–0.76)	0.74 (0.70–0.78)	0.75^*^ (0.72–0.80)	0.73 (0.69–0.78)	0.73 (0.68–0.78)
Bloom_OD_144	0.74 (0.52–0.96)	0.70 (0.66–0.74)	0.77 (0.72–0.81)	0.76 (0.71–0.81)	0.71 (0.66–0.78)	0.70 (0.63–0.77)	0.76 (0.71–0.81)
Berry_393	0.71 (0.43–0.99)	0.70 (0.66–0.74)	0.78 (0.74–0.82)	0.77 (0.73–0.81)	0.79^*^ (0.75–0.84)	0.77 (0.73–0.81)	0.79 (0.74–0.84)
Suliman_RISK_4	NA	0.62 (0.57–0.69)	0.62 (0.58–0.68)	0.55 (0.53–0.58)	0.74^**^ (0.70–0.79)	0.60 (0.55–0.66)	0.61 (0.56–0.66)
Zak_RISK_16	NA	0.62 (0.56–0.70)	0.85^***^ (0.81–0.88)	0.84^***^ (0.80–0.88)	0.83^***^ (0.80–0.86)	0.83^***^ (0.79–0.86)	0.84^***^ (0.80–0.88)
Leong_RISK_29	NA	0.68 (0.65–0.73)	0.58^*^ (0.56–0.61)	0.59^*^ (0.56–0.61)	0.75 (0.70–0.80)	0.60 (0.56–0.64)	0.61^*^ (0.57–0.65)

*: p-value < = 0.01, **: p-value < = 0.001, ***: p-value < = 0.0001 derived from Wilcoxon signed-rank test between the original model and corresponding gene set scoring methods; †: the original model results underperformed the Warsinske et al. by more than 0.05 units; ‡: the original model results outperformed the Warsiske et al. by more than 0.05 units

**Fig. 2:**
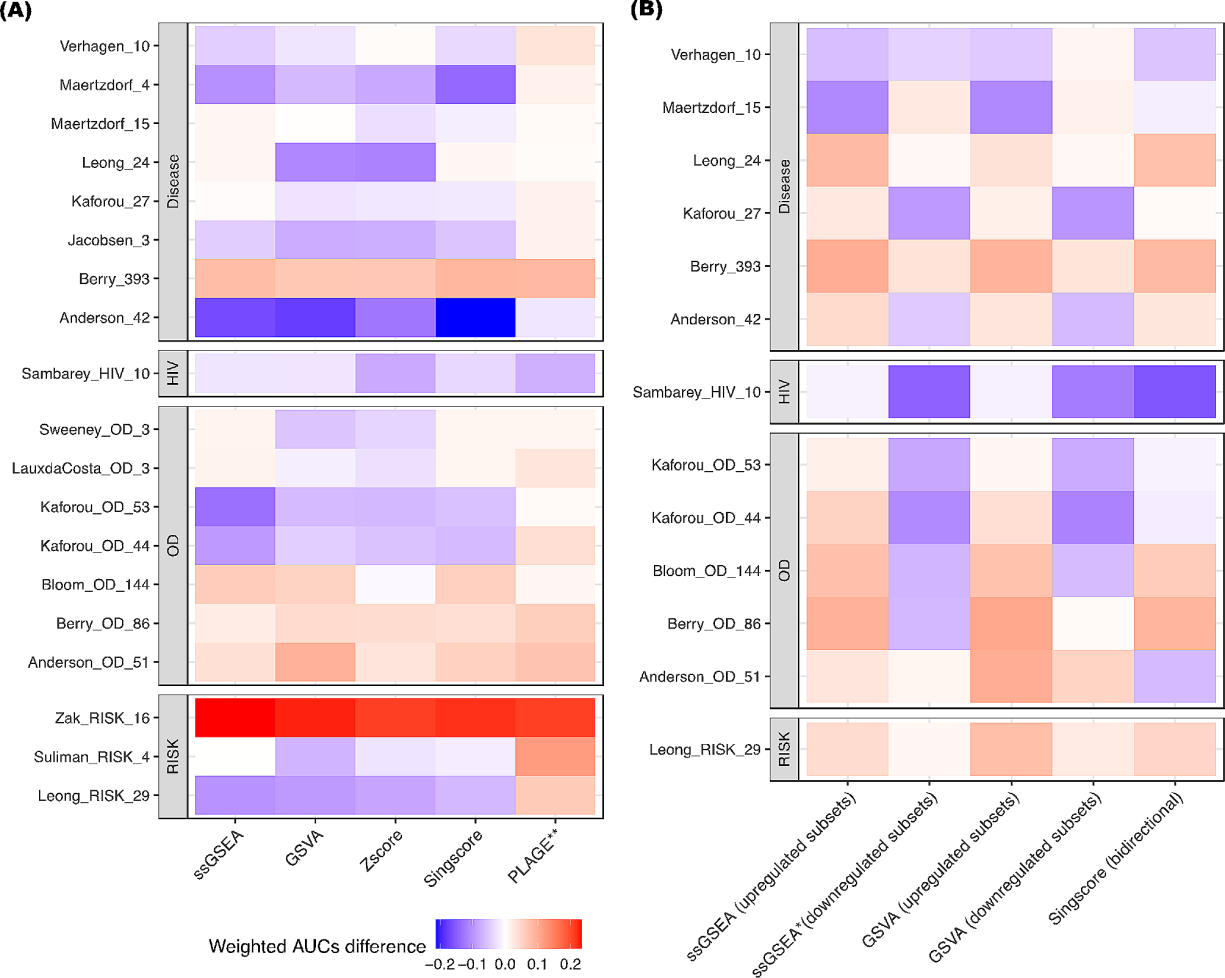
Differences in the weighted mean AUC values between the original model and the gene set scoring methods. Each grid showed the difference in the weighted mean AUC value between the corresponding gene set scoring methods (ssGSEA, GSVA, PLAGE, and Singscore) and the original models. The results from the original model were used as the baseline. For all 19 TB gene signatures, the weighted mean AUC results for ssGSEA, Singscore, GSVA, and Zscore were statistically equivalent to the results given by the original model (**A**). The weighted mean AUC results for ssGSEA and GSVA for the upregulated subsets of the gene signatures, and by Singscore (bidirectional scoring) and GSVA for the downregulated subsets of the gene signatures, were statistically equivalent to the results given by the original model (**B**). Red: the weighted mean AUC for gene scoring method *outperformed* the original model. Blue: the weighted mean AUC for gene scoring method *underperformed* the original model. *: p-value < 0.05 derived from the Wilcoxon signed-rank test. **: p-value < 0.01 derived from the same test

The weighted AUCs for Anderson_OD_51 computed by gene scoring methods surpassed that of its original model, although none of the results were statistically significant after adjusting for multiple testing (p-value > 0.01; Table 2). For Berry_393, the weighted AUCs computed from its original model underperformed all five gene scoring methods; specifically, the AUC computed by PLAGE was 0.79 (95% CI: 0.75–0.84), which was significantly higher (p-value < = 0.01) than that of the original model which had an AUC of 0.70 (95% CI: 0.66–0.74). The results from Zak_RISK_16 given by its original model also underperformed five gene scoring methods, and all the superior weighted AUCs were significant (p-value < = 1e-04) (Table 2). The outperformance of the weighted AUCs given by PLAGE was consistent in most of the gene signatures asides from Sambarey_HIV_10 and Anderson_42 (Fig. 2A). Except PLAGE (p-value < = 0.01), the results from the remaining gene scoring methods were statistically equivalent to the weighted AUCs given by the original model (p-value > 0.05; Fig. 2A).

### Accounting for gene expression direction

Following the implementation of the biomarker splitting strategy for gene signatures containing ten or more genes, we noted significant improvements in the weighted AUCs for the upregulated subsets of Berry_OD_86. Specifically, based on ssGSEA, the AUC was 0.78 (95% CI:0.75–0.82), while using GSVA resulted in an AUC of 0.80 (95% CI: 0.76–0.84). These values represent notable improvements compared to the original model (p-value < = 1e-04 for both methods; Table 3). Similar improvement was observed for the upregulated subsets of Berry_393 (p-value < = 0.01 for both methods; Table 3). Furthermore, the performance of Berry signatures, as evaluated by Singscore bidirectional scoring, surpassed that of their original models (p-value < = 1e-03 for both gene sets; Table 3). Interestingly, Anderson_OD_51 is the only gene signature for which the weighted AUCs from GSVA outperformed its original model for both its upregulated and downregulated subsets (AUC > = 0.77 and p-value < 0.05 for both subsets; Table 3). Overall, the weighted AUCs from all methods were statistically equivalent to the results given by the original model, except for the results computed from the signatures’ downregulated subset using ssGSEA (p-value = 0.036; Fig. 2B).

**Table 3:** Weighted mean AUC and 95% CI results for the upregulated and downregulated subsets of gene signatures using ssGSEA, GSVA, and Singscore bidirectional scoring methods

Signature	TBSignatureProfiler (Original Model)	TBSignatureProfiler (ssGSEA: upregulated)	TBSignatureProfiler (ssGSEA: downregulated)	TBSignatureProfiler (GSVA: upregulated)	TBSignatureProfiler (GSVA: downregulated)	TBSignatureProfiler (Singscore)
Verhagen_10	0.61 (0.57–0.66)	0.55 (0.54–0.58)	0.57 (0.55–0.62)	0.56 (0.53–0.60)	0.62 (0.58–0.67)	0.56 (0.54–0.59)
Sambarey_HIV_10	0.83 (0.76–0.87)	0.82 (0.78–0.85)	0.68 (0.64–0.73)	0.82 (0.78–0.85)	0.71 (0.65–0.76)	0.67^*^ (0.62–0.74)
Maertzdorf_15	0.82 (0.79–0.85)	0.71^*^ (0.67–0.76)	0.85 (0.81–0.88)	0.71^**^ (0.68–0.75)	0.84 (0.80–0.88)	0.80 (0.77–0.84)
Leong_24	0.72 (0.67–0.78)	0.80 (0.76–0.85)	0.73 (0.69–0.77)	0.76 (0.71–0.80)	0.73 (0.69–0.77)	0.80 (0.76–0.84)
Kaforou_27	0.81 (0.77–0.85)	0.84 (0.81–0.88)	0.72^***^ (0.68–0.76)	0.83 (0.80–0.87)	0.71^***^ (0.67–0.75)	0.82 (0.78–0.86)
Anderson_42	0.78 (0.73–0.83)	0.82 (0.78–0.86)	0.73^*^ (0.69–0.78)	0.81 (0.78–0.84)	0.72^*^ (0.68–0.76)	0.81 (0.77–0.85)
Kaforou_OD_44	0.76 (0.69–0.81)	0.81 (0.75–0.84)	0.65 (0.59–0.71)	0.80(0.74–0.84)	0.64 (0.59–0.70)	0.74 (0.67–0.80)
Anderson_OD_51	0.71 (0.64–0.78)	0.75 (0.70–0.80)	0.73 (0.70–0.76)	0.81 (0.78–0.84)	0.77 (0.71–0.82)	0.65^*^ (0.62–0.70)
Kaforou_OD_53	0.83 (0.78–0.87)	0.85 (0.81–0.88)	0.75^***^ (0.70–0.80)	0.84 (0.81–0.87)	0.76^***^ (0.70–0.80)	0.82 (0.78–0.86)
Berry_OD_86	0.69 (0.66–0.72)	0.78^***^ (0.75–0.82)	0.63 (0.60–0.68)	0.80^***^ (0.76–0.84)	0.70 (0.66–0.75)	0.78^**^ (0.74–0.82)
Bloom_OD_144	0.70 (0.66–0.74)	0.78 (0.74–0.83)	0.64 (0.60–0.68)	0.78 (0.73–0.83)	0.64^***^ (0.61–0.68)	0.77 (0.73–0.81)
Berry_393	0.70 (0.66–0.74)	0.80^*^ (0.76–0.85)	0.74 (0.70–0.79)	0.79^*^ (0.76–0.84)	0.74 (0.70–0.79)	0.79^**^ (0.75–0.84)
Leong_RISK_29	0.68 (0.65–0.73)	0.72 (0.69–0.77)	0.69 (0.64–0.75)	0.76 (0.72–0.80)	0.71 (0.66–0.75)	0.73^**^ (0.70–0.77)

*: p-value < = 0.01, **: p-value < = 0.001, ***: p-value < = 0.0001 derived from Wilcoxon signed-rank test

### Gene set scoring methods versus original models

Figure S3A-E showed the performance of gene signatures across multiple datasets after filtering some datasets. Detailed information regarding the criteria used to filter datasets is provided in the online data supplement. Generally, Verhagen_10 had poor performance when evaluated using its original model, Zscore, and Singscore (Figure S3). Similar mediocre performance was observed for Suliman_RISK_4, where no studies were selected using GSVA and Zscore, and GSE107994 was the only study selected by ssGSEA and its original model ($\left|\varDelta AUC\right|$ = 0.061; $\rho$ = 0.7; Figure S3A; Figure S3C-D). Additionally, no studies had AUCs greater than 0.8 for Leong_RISK_29 when evaluated using ssGSEA, GSVA, and Zscore (Figure S3A; Figure S3C-D). When comparing the performance between ssGSEA and original model, Kaforou_27, Maertzdorf_15, and Sweeney_OD_3 showed high diagnostic accuracy in similar studies ($oc$ for Kaforou_27 and Maertzdorf_15: = 1.00, $oc$ for Sweeney_OD_3: = 0.86; Figure S3A). Significantly positive Spearman’s rank correlations of the predicted scores were observed for Kaforou_27 (${\rho }_{w}$ = 0.78) and Sweeney_OD_3 (${\rho }_{w}$ = 0.86), while significantly negative correlations were shown for Maertzdorf_15 (${\rho }_{w}$ = -0.81; p-value < = 1e-04 for three signatures; Figure S3A).

Furthermore, Kaforou_27, Kaforou_OD_53, and LauxdaCosta_OD_3 had significantly negative Spearman’s rank correlations for the predicted results given by PLAGE and their original models (-0.92 < ${\rho }_{w}$ < -0.82; p-value < 1e-04; ${\left|\varDelta AUC\right|}_{w}$ < 0.03 for all three gene sets; Figure S3B). Conversely, two Maertzdorf signatures demonstrated strong positive Spearman’s rank correlations of the prediction scores and small AUC differences (p-value for Maertzdorf_4: = 0.06, p-value for Maertzdorf_15: = 0.02), with ${\rho }_{w}$ of 0.88 for Maertzdorf_4 and 0.94 for Maertzdorf_15 (p-value < 1e-05; Figure S3B).

When comparing the results given by GSVA and signatures’ original models, Anderson_OD_51, Berry_393, Berry_OD_86, Bloom_OD_144, and Zak_RISK_16 showed distinct prediction patterns (0.11 <= ${\rho }_{w}$ <= 0.47, ${\left|\varDelta AUC\right|}_{w}$ > 0.05 for all five gene sets; Figure S3C). Similar to that of ssGSEA, the resulting scores from Maertzdorf_15 (${\rho }_{w}$ = -0.78, ${\left|\varDelta AUC\right|}_{w}$ = 0.043) and Maertzdorf_4 (${\rho }_{w}$ = -0.63, ${\left|\varDelta AUC\right|}_{w}$ = 0.051) were negatively correlated with the results from the signatures’ original model, with a study overlap coefficient of 1.00 for both gene signatures (Figure S3C).

Based on the results from Zscore, the performance of Anderson_OD_51, Berry_393, Berry_OD_86, Bloom_OD_144, and Zak_RISK_16 were different when compared to the results from their original models (-0.0037 <= ${\rho }_{w}$ <= 0.65, ${\left|\varDelta AUC\right|}_{w}$ > 0.05 for all five gene sets; Figure S3D). Similarly, Maertzdorf_15 (${\rho }_{w}$ = -0.85, ${\left|\varDelta AUC\right|}_{w}$ = 0.044) and Maertzdorf_4 (${\rho }_{w}$ = -0.74, ${\left|\varDelta AUC\right|}_{w}$ = 0.084) had negatively correlated prediction scores given using Zscore and their respective original models (Figure S3D).

With Singscore unidirectional scoring, although more studies with high AUCs were selected when assessing Anderson_OD_51, Berry_393, Berry_OD_86, Bloom_OD_144, and Zak_RISK_16, the results from these signatures had weak to moderate weighted Spearman’s rank correlation (0.16 <= ${\rho }_{w}$ <= 0.55) and a large AUC difference (${\left|\varDelta AUC\right|}_{w}$ >= 0.11 for all five gene sets; Figure S3E). Sweeney_OD_3 (${\rho }_{w}$ = 0.85, p-value < 1e-04; ${\left|\varDelta AUC\right|}_{w}$ = 0.031, p-value = 0.75) was the only gene set for which the results from the Singscore could act as a proxy for its original models, except for study GSE34608 (Figure S3E).

When assessing gene signatures based on their upregulated subset, all selected studies had positive Spearman’s rank correlations between the predicted scores given by ssGSEA and original models (Figure S4A). The results from subsets of Kaforou_27 and Kaforou_OD_53 showed similar diagnostic features for ssGSEA and their original models (for both gene sets: ${\rho }_{w}$ > 0.80; p-value < = 1e-04; ${\left|\varDelta AUC\right|}_{w}$ < 0.030; $oc$ = 1.00; Figure S4A). When gene signatures were evaluated using their downregulated subsets, only Maertzdorf_15 had an absolute Spearman’s rank correlation greater than 0.80 (${\rho }_{w}$ = -0.89, p-value < = 1e-05), a small AUC difference (${\left|\varDelta AUC\right|}_{w}$ = 0.033, p-value = 0.010), and a study overlap coefficient of 1.00 (Figure S4B). Among the signatures, only Berry_393 (${\rho }_{w}$ = -0.62), Leong_24 (${\rho }_{w}$ = -0.64), and Verhagen_10 (${\rho }_{w}$ = 0.60) had more studies with high AUCs using ssGSEA compared to their original models (0.052 < ${\left|\varDelta AUC\right|}_{w}$ < 0.191; Figure S4B).

For the evaluation of signatures’ upregulated subsets, the predicted scores given by GSVA and original models were positively correlated for most of the signatures across datasets, except GSE34608 (Figure S4C). Kaforou_27 demonstrated the highest weighted Spearman’s rank correlation (${\rho }_{w}$ = 0.90, p-value < 1e-05) and the smallest AUC difference (${\left|\varDelta AUC\right|}_{w}$ = 0.020, p-value = 0.68) among the 12 gene sets, which presented an equivalent prediction pattern compared to its original model (Figure S4C). Conversely, the upregulated subset of Maertzdorf_15 was the only gene set where the original model outperformed GSVA, presenting a greater number of studies with high AUCs from its original method (${\rho }_{w}$ = 0.68, ${\left|\varDelta AUC\right|}_{w}$ = 0.094; Figure S4C). When gene signatures were evaluated with their downregulated subsets using GSVA, nine out of 13 gene sets had a greater number of studies with high AUCs based on the results from their original model (Figure S4D). The results from Maertzdorf_15, similar to those for the ssGSEA method, had the highest absolute weighted Spearman’s rank correlation (${\rho }_{w}$ = -0.88, p-value < = 1e-05) and the smallest absolute AUC difference (${\left|\varDelta AUC\right|}_{w}$ = 0.03, p-value = 0.20; Figure S4D).

Finally, most of the selected studies had positive correlations for the predicted scores given by Singscore bidirectional scoring and their original methods, except study GSE62525 from Bloom_OD_144 and GSE101705 from Verhagen_10 (Figure S4E). Both Kaforou_27 and Kaforou_OD_53 had a weighted Spearman’s rank correlation greater than 0.85 (p-value < 1e-05), an absolute AUC difference smaller than 0.020 (p-value > 0.05), and a study overlap coefficient of 1.00. These findings suggest that results for these gene signatures given by Singscore bidirectional scoring could act as a proxy for the results given by their original models (Figure S4E).

## Discussion

TB diagnostics are moving toward using blood-based biomarkers, but serious gaps remain in the analyses of these data. Here, we evaluated the performance of 19 TB gene signatures in distinguishing active TB from other clinical conditions using the original published model and five different gene set scoring methods across 24 transcriptomic studies. These datasets represent real-world heterogeneity concerning geographic regions, host and pathogen genetics, and clinical context [18]. Our results suggested that an original gene signature model’s predictive ability can be improved or recaptured using some gene set scoring methods.

The five gene set scoring methods used here belong to a class of methods that compute a gene set enrichment score for each sample using only the genes from a signature. However, some differences between methods are present. Gene set scoring methods, including ssGSEA, GSVA, and Singscore, are *single-sample* methods that rank genes in each sample individually by comparing the ranks of the signature genes with the ranks of non-signature genes in the sample. Additionally, the original models for the gene signatures Sweeney_OD_3, Kaforou_27, Kaforou_OD_44, Kaforou_OD_53, Anderson_42, and Anderson_OD_51 could also be characterized as single sample methods, which rely on the expression of upregulated and downregulated subsets of genes within gene sets (Table 1). These single-sample methods were more likely to produce robust scores for individual subjects, especially in studies with small sample sizes or heterogeneous disease subtypes. In contrast, PLAGE belongs to the class of *multi-sample* methods, which implements singular value decomposition (SVD) on the standardized gene expression profile of all subjects in the dataset [33, 34, 36]. Multi-sample methods (e.g., PLAGE, random forest, etc.) were susceptible to changes in sample composition. The results given by multi-sample methods may be irreproducible if the size of samples for different disease subtypes changes [36], which is known as “test set bias” [38]. In our study, PLAGE consistently outperformed other gene ser scoring methods for most signatures (Table 2; Fig. 2A). This superior performance is attributed to the selection of biologically meaningful genes based on prior knowledge, combined with SVD-like analysis, which together ensure high sensitivity and effective prioritization [39].

The weighted AUCs given by these single-sample methods were sometimes lower than the AUC from the original model for some signatures (Table 3). For these cases, the biomarker splitting strategy improved the signatures’ diagnostic ability, consistent with existing publications in other fields [33, 40]. Moreover, the improvement of weighted AUCs based on the upregulated subsets was more dominant when compared to the results from the downregulated subset (Table 3). This is likely because upregulated genes are usually immune-related, such as FCGR1A/B, GBP5/6, C1QB, SEPTIN4, and ANDKRD22 [20], which generate a clear signal in active TB and are features of the immune response to the disease [41]. The weighted AUCs from Zak_RISK_16 were consistently greater than 0.80 for five gene set scoring methods (Table 2), mainly due to the overexpression of all genes within Zak_RISK_16 relative to its discovery dataset, with a large number of genes being highly differentially expressed from the recent Leicester clinical phenotype groups [41].

The data preprocessing and training procedures are specialized and intractable for most TB gene signature discovery cases, which contributes to low generalizability in some biomarkers when evaluating their performance on independent datasets by implementing the original model. Both Berry_393 and Berry_OD_86 used the K-nearest neighbor (KNN) algorithm, which demonstrated high classification ability in their discovery studies but had poor results across multiple studies (Fig. 1). KNN clustering worked well when gene expression values were normalized to the median of each control group [10]; however, performing such normalization for transcriptomic datasets originating from different clinical conditions or different platforms is unrealistic. KNN classification also assumes that similar inputs share similar labels; however, data points tend to be close together in high-dimensional spaces [42]. Furthermore, the performance of Verhagen_10 was poor across independent studies based on the results given by its original diagnostic model but had AUC values equal to 1.00 in datasets GSE81746 and GSE41055 (Fig. 1). This is a sign of overfitting, a common problem using random forest, which relies on optimizing the tuning parameters [43]. In these and many other ways, a gene signature’s diagnostic accuracy may be underestimated by evaluating its performance using its original model.

For Kaforou_27, the results given by both PLAGE and ssGSEA were highly correlated (${\rho }_{w}$ from PLAGE: = -0.92, ${\rho }_{w}$ from ssGSEA: = 0.78), with a small AUC difference (${\left|\varDelta AUC\right|}_{w}$ from PLAGE: = 0.030, ${\left|\varDelta AUC\right|}_{w}$ from ssGSEA: = 0.032), and it had a study overlap coefficient of 1.00 when compared to its original model (Figure S3A-B). This is primarily because of the presence of 21 overexpressed genes within the Kaforou_27 gene set, which dominates the performance of the gene signatures (Figure S4A). We identified a similar situation for Maertzdorf_15, which had 12 downregulated genes within its gene set. Maertzdorf_15 and its downregulated subsets had consistently high negative Spearman’s rank correlations (-0.89 <= ${\rho }_{w}$ <= -0.78) for the prediction scores given by its original model and gene set scoring methods, except for PLAGE (${\rho }_{w}$ = 0.94) and Singscore bidirectional scoring (${\rho }_{w}$ = 0.86; Figure S3; Figure S4B, D-E).

Our study has several limitations. We only compared the performance of active TB disease versus all other disease states regardless of how TB was diagnosed, and we did not perform subgroup analysis stratified by different clinical conditions, such as age, region, or comorbidities. Additionally, some gene sets were trained on small datasets, and the reconstructed models may differ from those in the original publication, as some studies did not provide sufficient details to recreate the original training model. Instead, we followed the details outlined in the previously published comparison of Warsinske et al. [18]. Due to different naming sequencing platforms for transcripts, some genes within signatures were missing across multiple studies, and we could not accurately evaluate the biomarkers’ diagnostic ability in those cases. Although KNN imputation (see details in the online data supplement) was used to estimate the expression values for those missing genes in the validation study, the imputation process may have potentially led to bias. Our comparison would still be valid if we followed similar procedures to handle missing values across independent studies.

In conclusion, we showed that gene set scoring methods are effective for evaluating gene signature accuracy for comparing active TB disease versus other clinical conditions. In some cases, PLAGE outperformed the original models when considering signatures’ weighted AUCs. The ssGSEA, GSVA, and Singscore methods can also capture the diagnostic accuracy of gene signatures by taking the gene directional information within gene sets into account. Given the challenges associated with rebuilding or re-evaluating the signatures’ original biomarker model(s), gene set scoring methods could serve as a reliable alternative computational methodology to apply or perform comparisons of TB biomarkers.

### Electronic supplementary material

Below is the link to the electronic supplementary material.

Supplementary Material 1

Supplementary Material 2

## Data Availability

The datasets generated and/or analyzed during the current study are available at: https://bioconductor.org/packages/release/data/experiment/html/curatedTBData.html.
