# Supplementary material for: Comparison of gene set scoring methods for reproducible evaluation of tuberculosis gene signatures
Source: BMC Infect Dis. 2024 Jun 20;24:610. doi: 10.1186/s12879-024-09457-z (PMC11191245; doi:10.1186/s12879-024-09457-z)
Supplement: Supplementary file 2 — Supplementary Material 2 [file 12879_2024_9457_MOESM2_ESM.docx]

**Comparison of gene set scoring methods for reproducible evaluation of tuberculosis gene signatures**

Xutao Wang, M.Sc; Arthur VanValkenberg, Ph.D., Aubrey R. Odom Ph.D, Jerrold J. Ellner, M.D.; Natasha S. Hochberg, M.D., M.P.H.; Padmini Salgame, Ph.D; Prasad Patil, Ph.D; W. Evan Johnson, Ph.D

**Online data supplement**

Table of Contents

[Supplementary Tables 3](#_Toc125980491)

[Supplementary Table 1 3](#_Toc125980492)

[Supplementary Figures 5](#_Toc125980493)

[Supplementary Figure 1 5](#_Toc125980494)

[Supplementary Figure 2 6](#_Toc125980496)

[Supplementary Figure 3 7](#_Toc125980497)

[Supplementary Figure 4 8](#_Toc125980498)

[Materials and Methods 9](#_Toc125980499)

[Gene signatures and original diagnostic models used for comparison 9](#_Toc125980500)

[Gene set scoring methods 10](#_Toc125980501)

[Imputation of missing genes and removal of batch effects 11](#_Toc125980502)

[Selection of datasets used for comparing gene set scoring methods with original models 12](#_Toc125980503)

[Statistical Evaluation of model performance 13](#_Toc125980504)

# **Supplementary Tables**

**Supplementary Table 1.** Summary of AUC and 95% CI results from the gene signature’s discovery/training study (Warsinske et al. sections indicates the performance of gene sets based on its original model given by Warsinske et al.).

| **Signature** | **Clinical Comparison** | **Dataset Compared** | **Original Publication** | **Warsinske et al.** | **TBSignatureProfiler** | |
| --- | --- | --- | --- | --- | --- | --- |
|  |  |  | **AUC (95% CI)** | **AUC (95% CI)** | **AUC (95% CI) Warsinske et al.** | **AUC (95%CI) ssGSEA** |
| Sweeney_OD_3 | ATB vs. HCs | GSE19491✧ | 0.96 (0.94 - 0.98) | 0.96 (0.94 - 0.98) | 0.95 (0.91 - 0.99) | 0.96 (0.91 - 0.99) |
|  |  | GSE42834✧ | 1.00 (1.00-1.00) | 1.00 (1.00-1.00) | 1.00 (1.00 - 1.00) | 0.92 (0.83 - 0.98) |
|  | ATB vs. LTBI | GSE19491✧ | 0.93 (0.91 - 0.95) | 0.93 (0.91-0.95) | 0.92 (0.87 - 0.97) | 0.93 (0.87 - 0.97) |
|  |  | GSE37250 | 0.93 (0.91 - 0.94) | 0.93 (0.91-0.94) | 0.93 (0.90 - 0.95) | 0.90 (0.86 - 0.93) |
|  | ATB vs. OD | GSE19491✧ | 0.92 (0.89 - 0.94) | 0.92 (0.89-0.94) | 0.90 (0.84 - 0.95) | 0.92 (0.88 - 0.97) |
|  |  | GSE37250 | 0.87 (0.85 - 0.89) | 0.87 (0.85-0.89) | 0.87 (0.83 - 0.91) | 0.83 (0.78 - 0.87) |
|  |  | GSE42834✧ | 0.84 (0.80 - 0.88) | 0.84 (0.80-0.88) | 0.84 (0.79 - 0.90) | 0.78 (0.67 - 0.85) |
| Jacobsen_3 | ATB vs. LTBI | GSE19491✧ | N/A | 0.93 (0.91-0.95) | 0.93 (0.88 - 0.97) | 0.79 (0.71 - 0.86) |
| LauxdaCosta_OD_3 | ATB vs. OD | GSE42834✧ | 0.95* (0.88-1.00*) | 1.00 (1.00-1.00) | 1.00 (1.00-1.00) | 0.82 (0.76 - 0.88) |
| Maertzdorf_4 | ATB vs. HCs | GSE74092 | 0.98 (0.96 - 1.00) | 0.99 (0.99-1.00) | 1.00 (1.00-1.00) | 0.71 (0.64 - 0.79) |
| Sambarey_HIV_10 | ATB vs. (LTBI & HCs & OD) | GSE37250* | N/A | 0.89 (0.87-0.91) | 0.89 (0.85 - 0.91) | 0.79 (0.75 - 0.82) |
| Verhagen_10 | ATB vs. (LTBI & HCs) | GSE41055 | N/A | 1.00 (1.00-1.00) | 1.00 (1.00-1.00) | 0.85 (0.66 - 0.97) |
| Maertzdorf_15 | ATB vs. HC | GSE74092 | 0.99 (0.97 - 1.00) | 1.00 (1.00-1.00) | 1.00 (1.00 - 1.00) | 0.95 (0.92 - 0.97) |
| Leong_24 | ATB vs. LTBI | GSE101705 | 0.98 (0.98 - 0.98) | 1.00 (1.00-1.00) | 1.00 (1.00 - 1.00) | 0.95 (0.87 - 1.00) |
| Kaforou_27 | ATB vs. LTBI | GSE19491✧ | 0.98 (0.95 - 1.00) | 0.95 (0.93-0.97) | 0.96 (0.92 - 0.99) | 0.95 (0.90 - 0.98) |
| Anderson_42 | ATB vs. LTBI | GSE39940 | 0.98 (0.95 - 1.00) | 0.97 (0.96-0.98) | 0.98 (0.95 - 0.99) | 0.93 (0.88 - 0.96) |
| Kaforou_OD_44 | ATB vs. OD | GSE19491✧ | 0.95 (0.89 - 0.99) | 0.91 (0.89-0.94) | 0.93 (0.90 - 0.96) | 0.69 (0.60 - 0.77) |
| Anderson_OD_51 | ATB vs. OD | GSE39940 | 0.86 (0.77 - 0.94) | 0.89 (0.87-0.91) | 0.90 (0.87 - 0.94) | 0.80 (0.75 - 0.85) |
| Kaforou_OD_53 | ATB vs. (LTBI & HCs & OD) | GSE19491✧ | N/A | 0.92 (0.89-0.94) | 0.92 (0.88 - 0.96) | 0.71 (0.63 - 0.77) |

**Supplementary Table 1 (Continued)**

| **Signature** | **Clinical Comparison** | **Dataset Compared** | **Original Publication** | **Warsinske et al.** | **TBSignatureProfiler** | |
| --- | --- | --- | --- | --- | --- | --- |
|  |  |  | **AUC (95% CI)** | **AUC (95% CI)** | **AUC (95% CI) Warsinske et al.** | **AUC (95%CI) ssGSEA** |
| Berry_OD_86 | ATB vs. LTBI | GSE19491✧ | N/A | 0.97 (0.96-0.99) | 0.96 (0.92 - 0.98) | 0.90 (0.85 - 0.95) |
|  | ATB vs. HCs | GSE19491✧ | N/A | 1.00 (1.00-1.00) | 0.99 (0.98 - 1.00) | 0.89 (0.84 - 0.94) |
| Bloom_OD_144 | ATB vs. (OD & HCs) | GSE42834✧ | 0.91 (Not available) | 0.99 (0.98-1.00) | 0.97 (0.94 - 1.00) | 0.80 (0.71 - 0.87) |
| Berry_393 | ATB vs. LTBI | GSE19491✧ | N/A | 0.97 (0.96-0.99) | 0.96 (0.93 - 0.99) | 0.94 (0.89 - 0.97) |
|  | ATB vs. HCs | GSE19491✧ | N/A | 0.99 (0.98-1.00) | 0.99 (0.97 - 1.00) | 0.95 (0.91 - 0.98) |
| Leong_RISK_29 | Progressor vs. Non-progressor | GSE79362 (Baseline) | N/A | N/A | 0.96 (0.93 - 0.99) | 0.56 (0.51 - 0.69) |
| Zak_RISK_16 | Progressor vs. Non-progressor | GSE79362 (Total Time Period) | 0.74 (0.73 - 0.76) | N/A | 0.72 (0.61 - 0.82) | 0.77 (0.62 - 0.87) |
| Suliman_RISK_4 (Site-Specific) | Progressor vs. Non-progressor | GSE94438 | 0.67 (0.57-0.77) | N/A | 0.71 (0.65 - 0.77) | 0.61 (0.55 - 0.67) |

The original publication section presented the AUC and 95% CI for each signature as reported in their respective studies. The Warsinske et al. subsection showed results re-evaluated using the original models functionality implemented in the *TBSignatureProfiler*. The ssGSEA section presented the performance of each gene signature using ssGSEA from TBSignatureProfiler (Courtesy to Warsinske et al. work; ATB: active tuberculosis).

* Original training studies were not available for the signatures;

✧ Superseries of multiple datasets

# **Supplementary Figures**

**Supplementary Figure 1.** The distribution of AUC values for 19 TB gene signatures across 24 studies using ssGSEA **(A)**, GSVA **(B)**, PLAGE **(C)**, Zscore **(D)**, and Singscore unidirectional version **(E)**. The datasets are ordered the same as in Figure 1.

#

**Supplementary Figure 2**. The distribution of AUC values for **upregulated and downregulated subsets** of 13 TB gene signatures across 24 studies using ssGSEA **(A)**, GSVA **(B)**, and Singscore bidirectional version **(C)**. The datasets are ordered the same as in Figure 1.


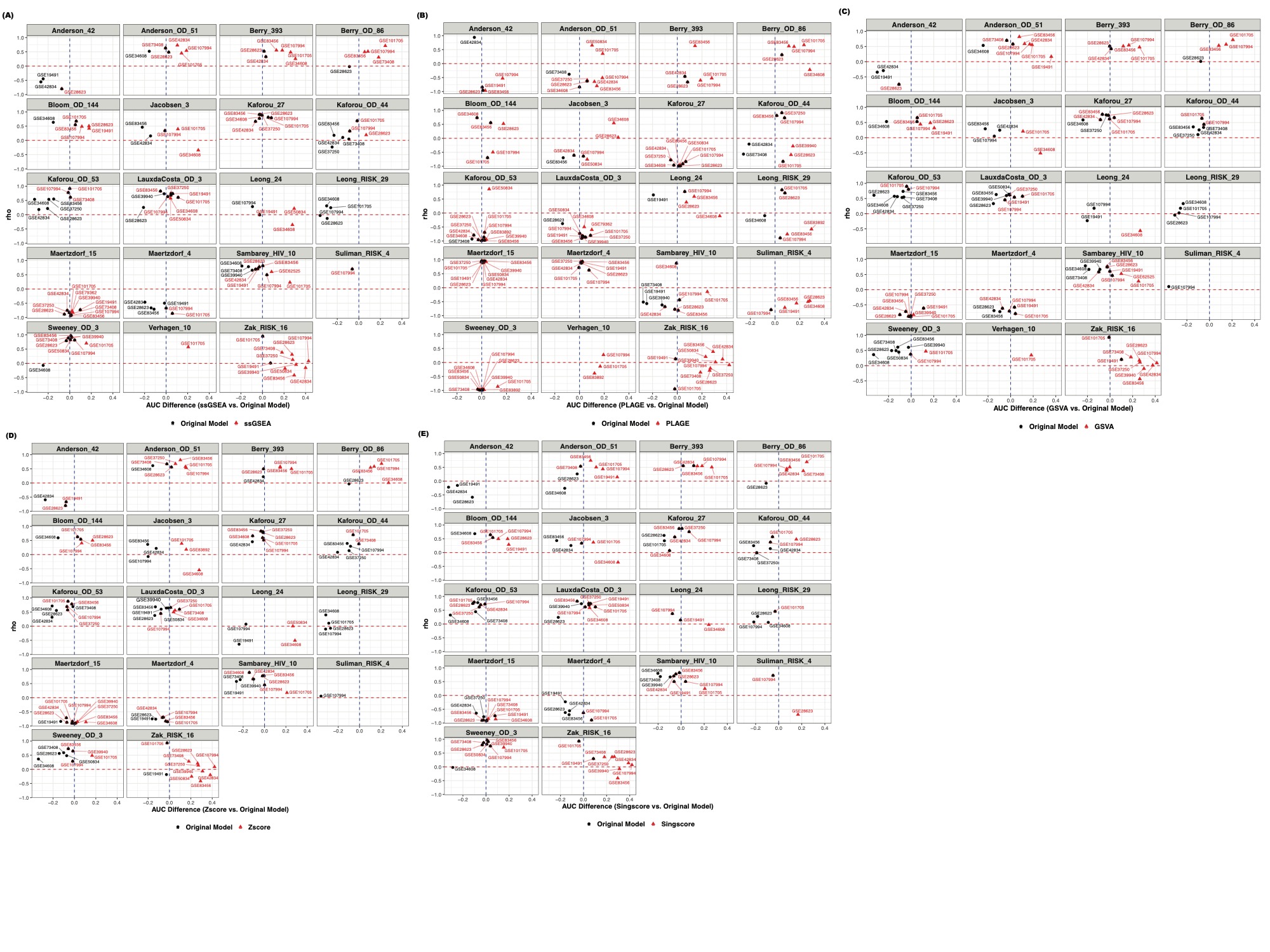


**Supplementary Figure 3.** Spearman’s rank correlation versus AUC difference for studies with sample size larger than 40 and computed AUC values greater than 0.8, based on the results derived from original models and ssGSEA **(A)**, original models and PLAGE **(B)**, original models and GSVA **(C)**, original models and Zscore **(D)**, and original models and Singscore (unidirectional version) **(E)**. Overlapping points indicate the same dataset(s).

**
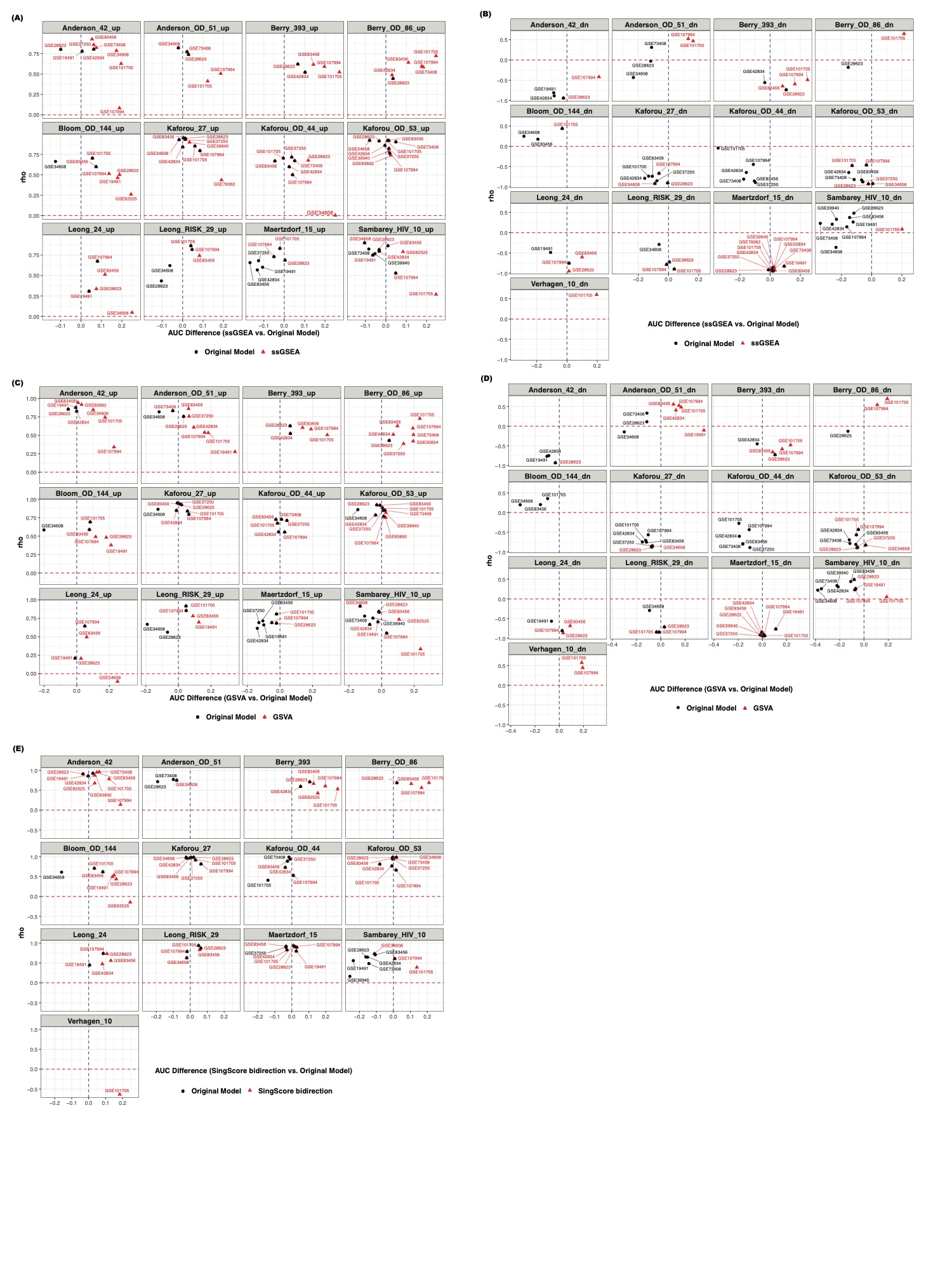
**

**Supplementary Figure 4**. Spearman’s rank correlation versus AUC difference for studies with sample size larger than 40 and computed AUC greater than 0.8, based on results derived from original models and ssGSEA (evaluated with upregulated subsets) **(A)**, original models and ssGSEA (evaluated with downregulated subsets) **(B)**, original models and GSVA (evaluated with upregulated subsets) **(C)**, original models and GSVA (evaluated with downregulated subsets) **(D)**, and original models and Singscore (bidirectional version) **(E)**. Overlapping points indicate the same dataset(s).

# **Materials and Methods**

# **Gene signatures and original diagnostic models used for comparison**

Nineteen existing TB gene signatures were selected for this study primarily based on the results of Warsinske *et al*. (Table 1) [1–14] to make a fair comparison for the performance of these signatures. Sixteen gene signatures were trained to predict active TB versus other clinical conditions. Three additional gene signatures that predict progression from LTBI to active TB disease, Zak_RISK_16 [12], Suliman_RISK_4 [13], and Leong_RISK_29 [14], were also included. Twenty-four datasets [2–5,8,11,15–34] that include subjects with active TB and other clinical conditions have been selected for evaluation. These datasets were selected in alignment with Warsinske *et al*. to systematically compare different gene scoring methods and the original models [35]. Twenty of these datasets were from microarray studies; the other four consisted of RNA-sequencing data. A detailed description of the transcriptomic studies included in this study can be found in the Supplementary Material.

The reconstruction of each signature’s original model followed the guidelines given by Warsinske et al. [35], who reconstructed these models based on information from each of the signature’s original publications. Two training methods were used for TB gene signature discovery: model-based and score-based approaches. The model-based approaches use machine learning algorithms such as support vector machines [36] or random forests [37] to reconstruct the biomarker’s original model. The score-based approaches primarily rely on the difference of sums or arithmetic means between the upregulated genes and the downregulated genes within the gene set, and therefore do not require model retraining. Overall, six out of the 19 identified gene signatures used score-based methods to distinguish active TB patients from other clinical conditions (Table 1). The reconstructed models were validated against the performance reported in the original publication using the AUC metric. To evaluate a biomarker’s ability to identify patients with active TB, the associated model was applied across the 24 curated transcriptomic studies.

## **Gene set scoring methods**

We used single sample GSEA (ssGSEA) [38], Gene Set Variation Analysis (GSVA) [39], Pathway level analysis of gene expression (PLAGE) [40], Zscore [41], and Singscore (unidirectional and bidirectional versions) [38,42] to evaluate the accuracy of TB gene signatures across 24 studies. The evaluation of distinct gene signatures using different gene set scoring methods and its original model was performed by the runTBSigProfiler function from the TBSignatureProfiler R package [20].

**Gene signature splitting strategy**

Some gene set scoring methods, including GSVA, ssGSEA, and Singscore, rank a signature’s genes against other genes in the dataset. Thus, gene sets with both upregulated and downregulated genes do not usually score well with these methods—they effectively counteract each other in the score computation. Most of the 19 signatures contain genes expressed strongly in both directions; thus, we proposed a ‘signature splitting’ strategy for gene signatures with equal to or more than ten genes to overcome this limitation. Gene signatures with less than ten genes were excluded here as their upregulated or downregulated gene subset was either empty or only contained one gene. In such cases, signature splitting would have a decreased effect on these gene sets compared to a large set.

For the ssGSEA and GSVA methods, we evaluated each signature’s performance using only the upregulated or the downregulated subsets of genes within the gene sets. For gene signatures that did not require reconstruction of the training model, the upregulated and downregulated genes were usually provided by the original publication. If unavailable in the original publication, the upregulated or the downregulated genes were identified by performing differential expression analysis on the datasets from which the associated biomarkers were derived using the R package DESeq2 [43] for RNA-seq data and limma [44] for microarray data. For the bidirectional implementation of Singscore, we used the simpleScore function from the singscore R package [42] to algorithmically combine the disease score for the upregulated and downregulated genes.

## **Imputation of missing genes and removal of batch effects**

Occasionally, various genes within gene sets were missing across transcriptomic studies, mainly due to the inconsistent gene coverage across platforms or different versions of the gene symbol naming systems across different sequencing platforms. This might affect a biomarker’s predictive accuracy in some datasets and across biomarker methods. Two steps were taken to mitigate this issue. First, we updated gene symbols for gene signatures and candidate datasets using the HGNChelper R package [45] to reduce gene misidentification during the training and validation process. More details on the matching of probe ID to gene symbol and missing gene information within each dataset can be found in the Supplementary Material. Second, a k-nearest neighbors (KNN) algorithm was applied to impute the missing gene expression values based on the signature’s discovery dataset(s) [46]. This procedure may elicit potential bias problems when large portions of genes within a signature are missing in some datasets. To make a fair comparison, we applied this imputation procedure to all biomarkers that used model-based diagnostic approaches across independent datasets. Functionality *reference ComBat* [47,48] was used to make generalized comparisons of signatures across the independent studies. *reference ComBat* removes batch effects between the training datasets and the testing datasets without changing the reference dataset’s gene expression values. With this functionality, the biomarker’s original model does not have to be retrained whenever the batch correction is performed or when the model is applied to a new dataset, increasing efficiency when evaluating biomarkers with model-based approaches.

## **Selection of datasets used for comparing gene set scoring methods with original models**

We noticed that there are several studies (i.e. GSE81746, GSE107331, GSE29536 etc.) with a small sample size (n < 40) which only consisted of healthy controls (HCs) and subjects with active TB. For these studies, most was generalizable to gene signatures that could distinguish patients with active TB phenotype with high predictive accuracy; however, such performance could not be generalized toward large populations or to cohorts with heterogeneous disease types including LTBI. To produce a fair comparison of gene signatures’ performance using gene set scoring methods and their original models, for each profiling method (including both gene set scoring methods and original models), we selected datasets with a sample size greater than 40 and AUC estimates greater than 0.80 across all TB biomarkers simultaneously excluding the corresponding training study for each biomarker.

## **Statistical Evaluation of model performance**

The AUC value corresponding to sample scores against disease subtypes for each TB gene signature was calculated for each dataset. The sample-size-weighted mean AUC (weighted AUCs) was used to assess the overall performance of each gene set across independent studies while excluding the discovery dataset(s) used to train the corresponding signature [35]. AUC results were calculated with the R package ROCit [49] compute estimates. Each signature’s 95% confidence interval for the weighted mean AUC value was calculated using the bootstrap method with 10,000 bootstrap resamples. The Wilcoxon paired test was applied to test whether AUCs given by different methods were significantly different from each other.

**REFERENCE FOR SUPPLEMENT**

1. Sweeney TE, Braviak L, Tato CM, Khatri P. Genome-wide expression for diagnosis of pulmonary tuberculosis: a multicohort analysis. Lancet Respir Med. 2016;4:213–24.

2. Kaforou M, Wright VJ, Oni T, French N, Anderson ST, Bangani N, et al. Detection of tuberculosis in HIV-infected and -uninfected African adults using whole blood RNA expression signatures: a case-control study. PLoS Med. 2013;10:e1001538.

3. Anderson ST, Kaforou M, Brent AJ, Wright VJ, Banwell CM, Chagaluka G, et al. Diagnosis of childhood tuberculosis and host RNA expression in Africa. N Engl J Med. 2014;370:1712–23.

4. Berry MPR, Graham CM, McNab FW, Xu Z, Bloch SAA, Oni T, et al. An interferon-inducible neutrophil-driven blood transcriptional signature in human tuberculosis. Nature. 2010;466:973–7.

5. Bloom CI, Graham CM, Berry MPR, Rozakeas F, Redford PS, Wang Y, et al. Transcriptional blood signatures distinguish pulmonary tuberculosis, pulmonary sarcoidosis, pneumonias and lung cancers. PLoS One. 2013;8:e70630.

6. Laux da Costa L, Delcroix M, Dalla Costa ER, Prestes IV, Milano M, Francis SS, et al. A real-time PCR signature to discriminate between tuberculosis and other pulmonary diseases. Tuberculosis . 2015;95:421–5.

7. Jacobsen M, Repsilber D, Gutschmidt A, Neher A, Feldmann K, Mollenkopf HJ, et al. Candidate biomarkers for discrimination between infection and disease caused by Mycobacterium tuberculosis. J Mol Med . 2007;85:613–21.

8. Leong S, Zhao Y, Joseph NM, Hochberg NS, Sarkar S, Pleskunas J, et al. Existing blood transcriptional classifiers accurately discriminate active tuberculosis from latent infection in individuals from south India. Tuberculosis . 2018;109:41–51.

9. Maertzdorf J, McEwen G, Weiner J 3rd, Tian S, Lader E, Schriek U, et al. Concise gene signature for point-of-care classification of tuberculosis. EMBO Mol Med. 2016;8:86–95.

10. Sambarey A, Devaprasad A, Mohan A, Ahmed A, Nayak S, Swaminathan S, et al. Unbiased Identification of Blood-based Biomarkers for Pulmonary Tuberculosis by Modeling and Mining Molecular Interaction Networks. EBioMedicine. 2017;15:112–26.

11. Verhagen LM, Zomer A, Maes M, Villalba JA, Del Nogal B, Eleveld M, et al. A predictive signature gene set for discriminating active from latent tuberculosis in Warao Amerindian children. BMC Genomics. 2013;14:74.

12. Zak DE, Penn-Nicholson A, Scriba TJ, Thompson E, Suliman S, Amon LM, et al. A blood RNA signature for tuberculosis disease risk: a prospective cohort study. Lancet. 2016;387:2312–22.

13. Suliman S, Thompson EG, Sutherland J, Weiner J 3rd, Ota MOC, Shankar S, et al. Four-Gene Pan-African Blood Signature Predicts Progression to Tuberculosis. Am J Respir Crit Care Med. 2018;197:1198–208.

14. Leong S, Zhao Y, Ribeiro-Rodrigues R, Jones-López EC, Acuña-Villaorduña C, Rodrigues PM, Palaci M, Alland D, Dietze R, Ellner JJ, Johnson WE. Cross-validation of existing signatures and derivation of a novel 29-gene transcriptomic signature predictive of progression to TB in a Brazilian cohort of household contacts of pulmonary TB. Tuberculosis. Churchill Livingstone; 2020;120:101898.

15. Banchereau R, Jordan-Villegas A, Ardura M, Mejias A, Baldwin N, Xu H, et al. Host immune transcriptional profiles reflect the variability in clinical disease manifestations in patients with Staphylococcus aureus infections. PLoS One. Public Library of Science (PLoS); 2012;7:e34390.

16. Park J, Munagala I, Xu H, Blankenship D, Maffucci P, Chaussabel D, et al. Interferon signature in the blood in inflammatory common variable immune deficiency. PLoS One. Public Library of Science (PLoS); 2013;8:e74893.

17. Obermoser G, Presnell S, Domico K, Xu H, Wang Y, Anguiano E, et al. Systems scale interactive exploration reveals quantitative and qualitative differences in response to influenza and pneumococcal vaccines. Immunity. Elsevier BV; 2013;38:831–44.

18. Singhania A, Verma R, Graham CM, Lee J, Tran T, Richardson M, et al. A modular transcriptional signature identifies phenotypic heterogeneity of human tuberculosis infection. Nat Commun. 2018;9:2308.

19. VanValkenburg A, Kaipilyawar V, Sarkar S, Lakshminarayanan S, Cintron C, Prakash Babu S, et al. Malnutrition leads to increased inflammation and expression of tuberculosis risk signatures in recently exposed household contacts of pulmonary tuberculosis. Front Immunol. 2022;13:1011166.

20. Johnson WE, Odom A, Cintron C, Muthaiah M, Knudsen S, Joseph N, Babu S, Lakshminarayanan S, Jenkins DF, Zhao Y, Nankya E, Horsburgh CR, Roy G, Ellner JJ, Sarkar S, Salgame P, Hochberg NS. Comparing Tuberculosis Gene Signatures in Malnourished Individuals using the TBSignatureProfiler. BMC Infect Dis. 2020;

21. Maertzdorf J, Ota M, Repsilber D, Mollenkopf HJ, Weiner J, Hill PC, et al. Functional correlations of pathogenesis-driven gene expression signatures in tuberculosis. PLoS One. Public Library of Science (PLoS); 2011;6:e26938.

22. Elliott TOJP, Owolabi O, Donkor S, Kampmann B, Hill PC, Ottenhoff THM, et al. Dysregulation of apoptosis is a risk factor for tuberculosis disease progression. J Infect Dis. Oxford University Press (OUP); 2015;212:1469–79.

23. Blankley S, Graham CM, Turner J, Berry MPR, Bloom CI, Xu Z, et al. The transcriptional signature of active tuberculosis reflects symptom status in extra-pulmonary and pulmonary tuberculosis. PLoS One. Public Library of Science (PLoS); 2016;11:e0162220.

24. Cai Y, Yang Q, Tang Y, Zhang M, Liu H, Zhang G, et al. Increased complement C1q level marks active disease in human tuberculosis. PLoS One. Public Library of Science (PLoS); 2014;9:e92340.

25. Esmail H, Lai RP, Lesosky M, Wilkinson KA, Graham CM, Horswell S, et al. Complement pathway gene activation and rising circulating immune complexes characterize early disease in HIV-associated tuberculosis. Proc Natl Acad Sci U S A. 2018;115:E964–73.

26. Maertzdorf J, Weiner J 3rd, Mollenkopf H-J, TBornotTB Network, Bauer T, Prasse A, et al. Common patterns and disease-related signatures in tuberculosis and sarcoidosis. Proc Natl Acad Sci U S A. Proceedings of the National Academy of Sciences; 2012;109:7853–8.

27. Walter ND, Miller MA, Vasquez J, Weiner M, Chapman A, Engle M, et al. Blood Transcriptional Biomarkers for Active Tuberculosis among Patients in the United States: a Case-Control Study with Systematic Cross-Classifier Evaluation. J Clin Microbiol. 2016;54:274–82.

28. Ottenhoff THM, Dass RH, Yang N, Zhang MM, Wong HEE, Sahiratmadja E, et al. Genome-wide expression profiling identifies type 1 interferon response pathways in active tuberculosis. PLoS One. Public Library of Science (PLoS); 2012;7:e45839.

29. Dawany N, Showe LC, Kossenkov AV, Chang C, Ive P, Conradie F, et al. Identification of a 251 gene expression signature that can accurately detect M. tuberculosis in patients with and without HIV co-infection. PLoS One. Public Library of Science (PLoS); 2014;9:e89925.

30. Marais S, Lai RPJ, Wilkinson KA, Meintjes G, O’Garra A, Wilkinson RJ. Inflammasome activation underlying central nervous system deterioration in HIV-associated tuberculosis. J Infect Dis. 2017;215:677–86.

31. de Araujo LS, Vaas LAI, Ribeiro-Alves M, Geffers R, Mello FCQ, de Almeida AS, et al. Transcriptomic biomarkers for tuberculosis: Evaluation of DOCK9. EPHA4, and NPC2 mRNA expression in peripheral blood. Front Microbiol. Frontiers Media SA; 2016;7:1586.

32. Zak DE, Penn-Nicholson A, Scriba TJ, Thompson E, Suliman S, Amon LM, et al. A blood RNA signature for tuberculosis disease risk: a prospective cohort study. Lancet. Elsevier BV; 2016;387:2312–22.

33. Lee S-W, Wu LS-H, Huang G-M, Huang K-Y, Lee T-Y, Weng JT-Y. Gene expression profiling identifies candidate biomarkers for active and latent tuberculosis. BMC Bioinformatics. Springer Science and Business Media LLC; 2016;17 Suppl 1:3.

34. Tientcheu LD, Maertzdorf J, Weiner J, Adetifa IM, Mollenkopf H-J, Sutherland JS, et al. Differential transcriptomic and metabolic profiles of M. africanum- and M. tuberculosis-infected patients after, but not before, drug treatment. Genes Immun. Springer Science and Business Media LLC; 2015;16:347–55.

35. Warsinske H, Vashisht R, Khatri P. Host-response-based gene signatures for tuberculosis diagnosis: A systematic comparison of 16 signatures. PLoS Med. 2019;16:e1002786.

36. Noble WS. What is a support vector machine? Nat Biotechnol. 2006;24:1565–7.

37. Pavlov YL. Random Forests. Walter de Gruyter GmbH & Co KG; 2019.

38. Subramanian A, Tamayo P, Mootha VK, Mukherjee S, Ebert BL, Gillette MA, et al. Gene set enrichment analysis: a knowledge-based approach for interpreting genome-wide expression profiles. Proc Natl Acad Sci U S A. 2005;102:15545–50.

39. Hänzelmann S, Castelo R, Guinney J. GSVA: gene set variation analysis for microarray and RNA-seq data. BMC Bioinformatics. 2013;14:7.

40. Tomfohr J, Lu J, Kepler TB. Pathway level analysis of gene expression using singular value decomposition. BMC Bioinformatics. 2005;6:225.

41. Lee E, Chuang H-Y, Kim J-W, Ideker T, Lee D. Inferring pathway activity toward precise disease classification. PLoS Comput Biol. 2008;4:e1000217.

42. Foroutan M, Bhuva DD, Lyu R, Horan K, Cursons J, Davis MJ. Single sample scoring of molecular phenotypes. BMC Bioinformatics. 2018;19:404.

43. Love MI, Huber W, Anders S. Moderated estimation of fold change and dispersion for RNA-seq data with DESeq2. Genome Biol. 2014;15:550.

44. Ritchie ME, Phipson B, Wu D, Hu Y, Law CW, Shi W, et al. limma powers differential expression analyses for RNA-sequencing and microarray studies. Nucleic Acids Res. Oxford Academic; 2015;43:e47–e47.

45. Oh S, Abdelnabi J, Al-Dulaimi R, Aggarwal A, Ramos M, Davis S, et al. HGNChelper: identification and correction of invalid gene symbols for human and mouse. F1000Res. F1000 Research Ltd; 2020;9:1493.

46. Hastie T, Tibshirani R, Narasimhan B, Chu G. impute: Imputation for microarray data [Internet]. 2020. Available from: 10.18129/B9.bioc.impute

47. Zhang Y, Jenkins DF, Manimaran S, Johnson WE. Alternative empirical Bayes models for adjusting for batch effects in genomic studies. BMC Bioinformatics. 2018;19:262.

48. Johnson WE, Li C, Rabinovic A. Adjusting batch effects in microarray expression data using empirical Bayes methods. Biostatistics. 2007;8:118–27.

49. Khan MRA, Brandenburger T. ROCit: Performance Assessment of Binary Classifier with Visualization [Internet]. 2020. Available from: https://CRAN.R-project.org/package=ROCit
